# Low carbohydrate and psychoeducational programs show promise for the treatment of ultra-processed food addiction: 12-month follow-up

**DOI:** 10.3389/fpsyt.2025.1556988

**Published:** 2025-04-14

**Authors:** Jen Unwin, Christine Delon, Heidi Giæver, Clarissa Kennedy, Molly Painschab, Frida Sandin, Charlotte Schon Poulsen, David A. Wiss

**Affiliations:** ^1^ The Collaborative Health Community, Oxford, United Kingdom; ^2^ Sweet Sobriety, Belgrade, MT, United States; ^3^ Levasockerfri, Linkoping, Sweden; ^4^ Nutrition in Recovery LLC, Los Angeles, CA, United States

**Keywords:** addiction, sugar, ultra-processed food, low carbohydrate diet, ketogenic diets

## Abstract

The topic of ultra-processed food addiction has been the subject of many peer-reviewed publications. Although on average 14% of adults may meet the criteria for ultra-processed food addiction in prevalence studies, it is not a recognized clinical diagnosis, hence a lack of published evidence-based treatment protocols and outcome data. In 2022, we reported outcomes pre- and post-intervention from an online, real food-based, low-carbohydrate educational program with psychosocial support related to ultra-processed food addiction recovery. The intervention was delivered across three locations, offering a common approach. The programs comprised weekly online sessions for 10–14 weeks, followed by monthly support groups. The previously published data were outcomes relating to ultra-processed food addiction symptoms measured by the modified Yale Food Addiction Scale 2.0, ICD-10 symptoms of substance use disorder related to food (CRAVED), and mental well-being as measured by the short version of the Warwick Edinburgh Mental Wellbeing Scale, pre- and post-intervention. The current report focuses on the same cohort’s 6- and 12-month follow-up data. The 12-month follow-up data show significant, sustained improvement in ultra-processed food addiction symptoms and mental well-being. These data are the first long-term follow-up results to be published for a food addiction program. Research is now needed to evaluate and compare other long-term interventions for this impairing and increasingly prevalent biopsychosocial condition.

## Graphical Abstract

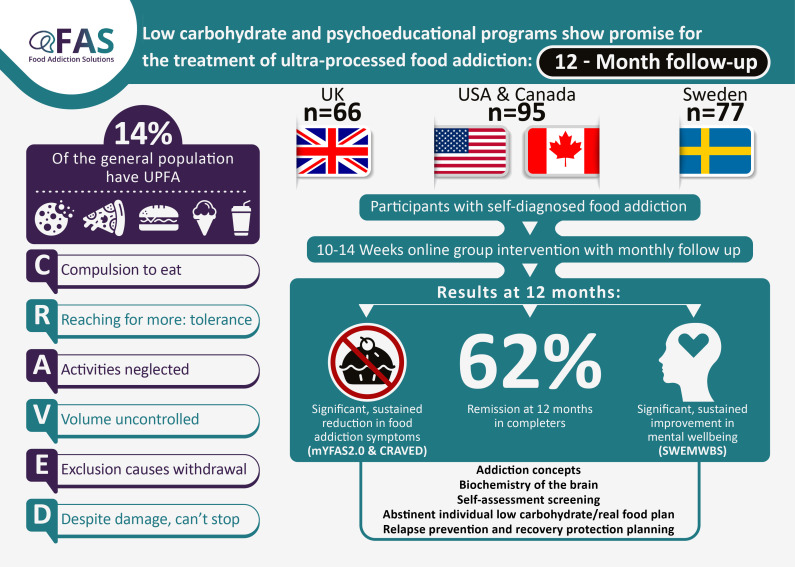

## Introduction

The term food addiction (FA) first appeared in the scientific literature in 1956 (1), and the number of papers on the topic continues to flourish. Debate has continued regarding a possible diagnosis (2–4), but FA has not yet been classified in the Diagnostic and Statistical Manual of Mental Disorders (5) (DSM-5) or the International Classification of Diseases (6) (ICD-10). Discussion also continues about the naming of this proposed disorder. A recent consensus exercise between international clinicians and academics in the field concluded overwhelmingly that the term ultra-processed food addiction (UPFA) most appropriately describes this disorder (7). Consequently, this report uses UPFA to refer to dependency behaviours related to added sugar, refined carbohydrates, and ultra-processed foods (8).

The most common operationalization of UPFA has been the Yale Food Addiction Scale (YFAS), initially published in 2009 (9), and the updated YFAS 2.0 (10). In terms of the most problematic foods for people with UPFA, Schulte et al. (2015) reported pizza, chocolate, chips (crisps), cookies (biscuits), and ice cream as key culprits (11). There are now several articles describing UPFA symptoms (12), the prevalence of the disorder (13), and purported mechanisms (14, 15) in both animal and human populations. The worldwide prevalence of UPFA in reported studies is ∼14%. UPFA is associated with increased BMI and eating disorders (16).

The 11 criteria for substance use disorder (SUD) from the DSM-5 (5) can be applied to processed foods high in combinations of refined carbohydrates/sugar, fat, and salt. Two or three symptoms would indicate mild UPFA, four or five would be moderate, and six or more would indicate severe UPFA. The criteria include:

- Consuming the substance in larger amounts or for longer than intended.
- Efforts to cut down or stop using the substance but not managing to.
- Time spent getting, using, or recovering from the substance.
- Cravings and urges to use the substance.
- Not managing to perform at work, home, or school because of substance use.
- Continuing to use the substance despite causing problems in relationships.
- Giving up important social, occupational, or leisure activities because of substance use.
- Using the substance repeatedly despite harmful consequences.
- Continuing to use the substance despite physical or psychological problems caused or worsened by the substance.
- Needing more of the substance to get the desired effect.
- Development of withdrawal symptoms, which are relieved by consumption of substance.

There are six criteria from the ICD-10 for SUD (6) where three or more symptoms indicate SUD:

- “Craving”, a strong desire or urge to use the substance.
- Difficulty controlling the onset, duration, amount, and termination of substance use.
- Increasing priority of substance use over other activities over time.
- Increased tolerance and the need to increase consumption over time.
- Physiological features of withdrawal when trying to abstain.
- Continued use of the substance despite mental or physical harm.

An addiction model of increased BMI and lifestyle-related diseases might explain why individuals struggle to comply with advice such as “eating in moderation”. An understanding of the addiction model of eating behavior reduces stigma by changing the blame narrative away from personal responsibility (17, 18). According to a recent study, 60% of professionals reported an interest in receiving training in addictive eating (19). An unmet need clearly exists.

There is a close association between UPFA and eating disorders (EDs), leading to calls for ED and UPFA addiction screening to complement one another (20). Individuals with bulimia nervosa (BN) have the highest prevalence of UPFA (48–95%), with binge eating disorder (BED; 55–80%), and anorexia nervosa (AN; 44–70%) also significantly associated (15, 18, 20–22). It has been suggested that increases in self-reported UPFA scores may be inflated by efforts to restrain eating, engage in compensatory behaviours (e.g., purging), or maintain lower body weights (8). Among adolescents, UPFA symptoms are an antecedent to dietary restraint (23). Meanwhile, UPFA symptoms can exist independently of ED symptoms and, therefore, should be considered as a distinct disorder warranting targeted evidence-based interventions (8, 20).

UPFA has been the focus of several neurobiological theories. Wiss et al. (2018) stated that “evidence is accumulating on the overlap of neural circuitry and commonalities between drug abuse and FA in humans” (14). Similarly, Lindgren et al. (2018) conceptualized UPFA *via* overlapping neural mechanisms with drug and alcohol addiction; specifically, a dampening of dopamine signaling and downregulation of the μ-opioid receptor, “coupled with impairment of prefrontal regions that are involved in inhibitory control” (15). There is a complex interaction between neurobiology and the hormones regulating eating. Lindgren et al. point to the challenge of UPFA treatment because, unlike other SUDs, total abstinence from food is not an option (15). However, abstinence from ultra-processed foods is entirely feasible. There have been no reports of addictive behaviours with real food proteins and fats.

Several interventions for UPFA have been suggested: medications (24), cognitive behavioral therapy (25), and brain stimulation, although published data is lacking. Psychoeducation improved UPFA severity, but a 73% prevalence post-intervention was still present (26). A study of 44 people undergoing bariatric surgery reduced UPFA prevalence from 32% to 2% at 6 months without further follow-up (27). A recent paper found that bariatric surgery had the most evidence of effectiveness (28). An energy-reduced diet in 11 people with BMIs above 30 (obesity) with symptoms of UPFA was found to normalize brain activation compared to people with high BMIs without FA (29). However, the follow-up was only 3 months, and no diet details were provided. Probiotics (30) and “infra-slow” brain training (31) have also been proposed. Reche-Garcia et al. (2024) found that studies looking at UPFA treatment were not of high quality and that there was a dearth of research on adolescent populations to date (28).

In a randomized controlled trial, Leary et al. (2024) demonstrated that participants with UPFA encouraged to set their own goals could increase dietary quality and that consumption of more nutrient-dense foods correlated with reduced UPFA symptoms (32).

Low-carbohydrate approaches have been suggested as having the potential to reduce UPFA symptoms (33). Ultra-processed, refined, or high-glycemic carbohydrates are a possible “trigger” mediating a neurochemical reward response similar to that seen in other addictions. The carbohydrate-insulin model of weight regulation supports observations of these foods triggering aberrant blood sugar and insulin spikes, subsequently leading to changes in metabolic and neurobiological signaling (34). A case series of three patients with BMI>30, BED, and UPFA managed over 6–7 months on a low-carbohydrate ketogenic approach demonstrated that binge eating and UPFA symptoms improved, accompanied by a 10–24% body weight loss, followed over 9–17 months (35).

Interventions for UPFA should investigate long-term sustainable improvements in symptoms and mental well-being, as well as the risk of developing new disorders of eating. UPFA treatment should not overemphasize weight loss, which can obscure the focus on the behavioral outcomes of the disorder (8). Given the increase in attention to weight stigma, clinicians and researchers may favor a more weight-inclusive approach (36).

In 2022, a poll of an online food addiction professional group (The Food Addiction Professionals Network) found that 20 out of 25 practitioners recommended carbohydrate-restricted food plans (unpublished data). This supports carbohydrate reduction as a common clinical practice for the management of UPFA. Other practitioners reported including more whole grains and fruit in their recommendations, and some preferred to assess clients individually. To our knowledge, no previous audits of long-term practice outcomes in UPFA have been published. The current audit describes the 6- and 12-month follow-up data from treatment services across three different countries offering online group interventions for people self-identifying as having UPFA, including an “abstinent” low carbohydrate “real food” approach and biopsychosocial education focused on addiction recovery. The pre- and post-program results have previously been published (37).

## Materials and methods

Three clinics in two continents [the United Kingdom (UK); North America (NA); Sweden (SE)], using online programs for treating people with UPFA, collaborated to implement the same screening instruments for intake and follow-up. The ethics protocol for the National Health Service in the UK was reviewed and indicated that a formal ethical review was not required as the project was an audit of pre-existing routine practice, and participants were self-referred.

### Participants

Social media and mailing list advertisements by the authors led to participant recruitment. Clinicians screened potential participants through online interviews to confirm self-identified UPFA symptoms. None of the programs accepted people under 18 years of age, pregnant, having severe mental health problems requiring ongoing specialist psychiatric support, or any doctor requesting exclusion. Each participant was given information about the program and audit process and had the opportunity to ask questions. Participants completed a consent form as part of the initial data collection to affirm that their anonymized data could be used to evaluate the programs. A unique code identified participants’ data to ensure anonymity. An information sheet (UK) and protocols are included as Supplementary Materials to the original publication (37). Participants paid a reduced fee (NA, SE) or voluntary donation (UK) to participate.

### Power calculation

Power calculations using the primary outcome measures of the mYFAS 2.0 (38) and the short version of the Warwick Edinburgh Mental Wellbeing Scale (39) (SWEMWBS) indicated that 26 participants were needed to complete the 1-year follow-up in each location, for a total of 78 total participants. Each location aimed to have 60–70 participants complete baseline data to ensure adequate numbers at 1-year follow-up. The total sample size at the start of the audit was 238 (UK=66, NA=95, SE=77), and at 12 months was 117 participants (UK=44, NA=50, SE=23).

### Measures

The mYFAS 2.0 is a short version of the YFAS 2.0 (38). The mYFAS 2.0 includes 13 items: one for each of the 11 UPFA criteria in the DSM-5 for SUD and two for assessing clinically significant impairment or distress. One example item is: “I ate until I was physically ill.” There are eight frequency choices on a scale from never to every day. The mYFAS 2.0 has good reliability and convergent and discriminant validity (38). The scale can be analyzed as a total number of criteria met (0–11, reported here) or categorically as an indication of diagnosis and severity.

A brief screening tool for UPFA symptoms based on the six ICD-10 criteria for SUD (6) was developed by HG and JU as a simple tool for clinicians. CRAVED, which has not been formally validated, is described and included in the Supplementary Materials to the original paper (37). Participants were asked to rate whether they had experienced the symptom in the last month (yes or no, possible score 0–6). An example item is: “I had such a strong desire or sense of compulsion at the thought of eating these foods, that I could not resist the urge to eat them.” A score of 3 or more out of 6 indicates a potential SUD according to ICD-10 (6).

The SWEMWBS is a short version of the Warwick-Edinburgh Mental Wellbeing Scale (39). The scale was developed to monitor mental well-being in the general population and evaluate programs designed to improve mental well-being. There are seven statements relating to functioning (e.g., I’ve been thinking clearly) with five Likert-type responses from none of the time to all the time. The measure has good construct and external validity and test-retest reliability (39). Scores range from 7-35, with higher scores indicating more positive well-being. A representative sample from England reported a mean of 23.6 (40).

The following data were also collected: age, gender, and body mass index (BMI). The online survey took approximately 10 minutes to complete.

### Programs

The programs consisted of 10–14 weeks of 90–120-minute sessions in groups of 11–40 participants. Some variation is due to each location having its own program materials and methods. Sessions consisted of educational content delivered live or pre-recorded, coaching discussions, and assigned reflections. The content of the programs included: understanding addiction concepts and biochemistry, self-assessment screening and reflection, abstinent low-carbohydrate individualized “real food” plan, imagining life beyond UPFA, new habits and tastes, resilience, relapse prevention planning, and personal lifestyle planning. Abstinence from sugar, grains, processed food, and any foods the individual participants reported being unable to moderate (e.g., peanut butter) was emphasized. A comparison of the three group programs and an example food plan (UK) are included in the Supplementary Materials of the 2022 paper (37). Following the active program phase, participants joined a monthly 60-minute clinician-facilitated online support group, which continued for 12 months. All groups also independently established additional social support through group chats and online meetings.

### Data collection and analysis

Data collection points were scheduled before and after the online group sessions and at 6 and 12 months thereafter. Participants entered their data into online forms, which were analyzed using R v4.4.0. Data were prepared using the tidyr package, version 1.3.1 (41). *P*-values were calculated using the Wilcoxon rank sum test with continuity correction, with p<0.05 considered statistically significant. Change over time was modelled with linear mixed models using the 1me4 package, version 1.1.35.3 (42), and p-values were estimated using 1merTest and anova Kenward_Roger approximation. All available data points for each time point were included in the analysis. Summary statistics were calculated using random effects models and the DerSimonian-Laird estimate (43) and visualized as forest plots using the meta package, version 7.0.0 (44).

## Results

Not all participants were available for follow-up, and a small number of participants who completed follow-up data could not be matched to baseline data because they entered unidentifiable codes. There were 44, 49, and 23 sets of matched data for UK, NA, and SE, respectively. **Table 1** shows the demographic and outcome variables for the participants across the three locations. Participants were predominantly female (91% UK, 97% NA, and 100% SE). Mean age at start was 52 (SD 11) (UK), 47 (SD 9.3) (NA) and 50 (SD 11) (SE).

**Table 1:** Age and outcome variables at all time points for each location.

Variable	UK				Sweden (SE)				North America (NA)			
	Before	After	Six months	12 months	Before	After	Six months	12 months	Before	After	Six months	12 months
Age at start	*52 (11) †66	-	-	-	*46 (9.3) †77	-	-	-	48 (11) †95	-	-	-
CRAVED	5.0 (4.0, 6.0) †66	3.0 (2.0, 5.0) †59 **p<0.0001**	4.0 (1.2, 4.8) †50 **p<0.0001**	3.0 (0.0, 5.0) †44 **p<0.0001**	5.0 (5.0, 6.0) †77	4.0 (2.0, 5.0) †53 **p=0.0001**	4.0 (1.0, 5.0) †41 **p=0.0007**	3.0 (0.0, 4.0) †23 **p=0.004**	5.0 (4.0, 6.0) †95 **p<0.0001**	3.0 (1.0, 5.0) †59 **p<0.0001**	3.5 (0.0, 4.0) †48 **p<0.0001**	3.0 (1.0, 4.8) †50 **p<0.0001**
mYFAS Diagnostic	6.5 (3.0, 10) †66	4.0 (0.0, 7.5) †59 **p=0.003**	1.0 (0.0, 3.8) †50 **p<0.0001**	2.0 (0.0, 5.0) †44 **p<0.0001**	8.0 (5.0, 10) †77	2.0 (0.0, 8.0) †53 **p<0.0001**	3.0 (1.0, 7.0) †41 **p<0.0001**	2.0 (0.0, 4.5) †23 **p=0.0014**	8.0 (5.0, 10) †95	7.0 (1.5, 10) †59 **p=0.0021**	1.0 (0.0, 4.2) †48 **p<0.0001**	1.0 (0.0, 5.5) †50 **p<0.0001**
SWEMWBS	20 (19, 22) †66	22 (20, 25) †59 **p<0.0001**	21 (19, 24) †50 **p=0.028**	22 (19, 25) †44 **p= 0.001**	20 (19, 23) †77	23 (21, 25) †53 **p<0.0001**	23 (19, 25) †41 p<0.18	23 (21, 25) †23 **p=0.0016**	21 (19, 22) †95	23 (22, 25) †59 **p<0.0001**	22 (22, 25) †48 **p<0.0001**	23 (20, 25) †50 **p=0.0017**
BMI	32 (28, 38) †66	31 (26, 36) †58 **p=0.010**	31 (28, 38) †49 p=0.492	30 (26, 35) †43 **p= 0.031**	28 (26, 33) †77	28 (25, 33) †51 **p=0.002**	28 (26, 32) †41 p=0.829	28 (24, 31) †23 p=0.649	31 (24, 38) †90	32 (25, 38) †55 **p=0.0005**	32 (26, 38) †44 p=0.1503	29 (24, 36) †48 **p=0.0077**
Key	Median (IQR) *mean (sd) or †N	Median (IQR) *mean (sd) †N			Median (IQR) *mean (sd) or †N	Median (IQR) *mean (sd) †N			Median (IQR) *mean (sd) or †N	Median (IQR) *mean (sd) †N		

Significant results shown in bold.

* = Mean (SD).

† = N.

Reductions in UPFA symptoms over time were significant across both mYFAS 2.0 and CRAVED across all locations (all p-values < 0.005). Improvements in mental well-being (SWEMWBS) were significant at 12 months for all locations (all p-values < 0.002). Improvements in BMI were significant at 12 months in the UK (p=0.031) and NA (p=0.007)

**Figure 1:**
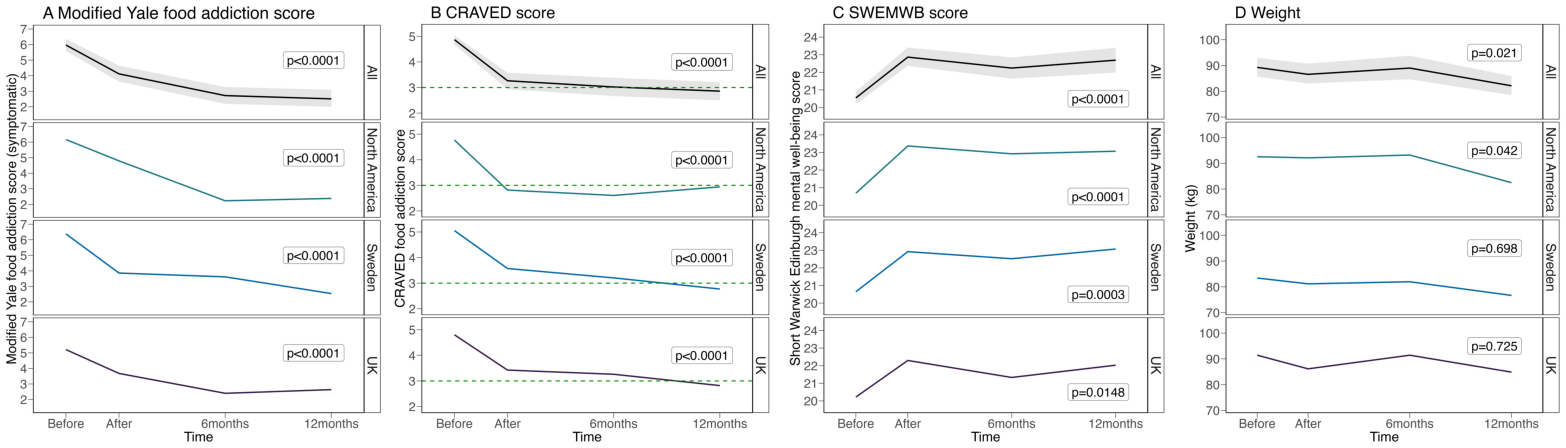
**(A)** mYFAS 2.0, **(B)** CRAVED, **(C)** SWEMWBS & **(D)** weight scores over time for all locations.

**Figure 1** displays significant improvements in mYFAS 2.0, CRAVED and SWEMWBS scores over time for all locations.

**Figure 2:**
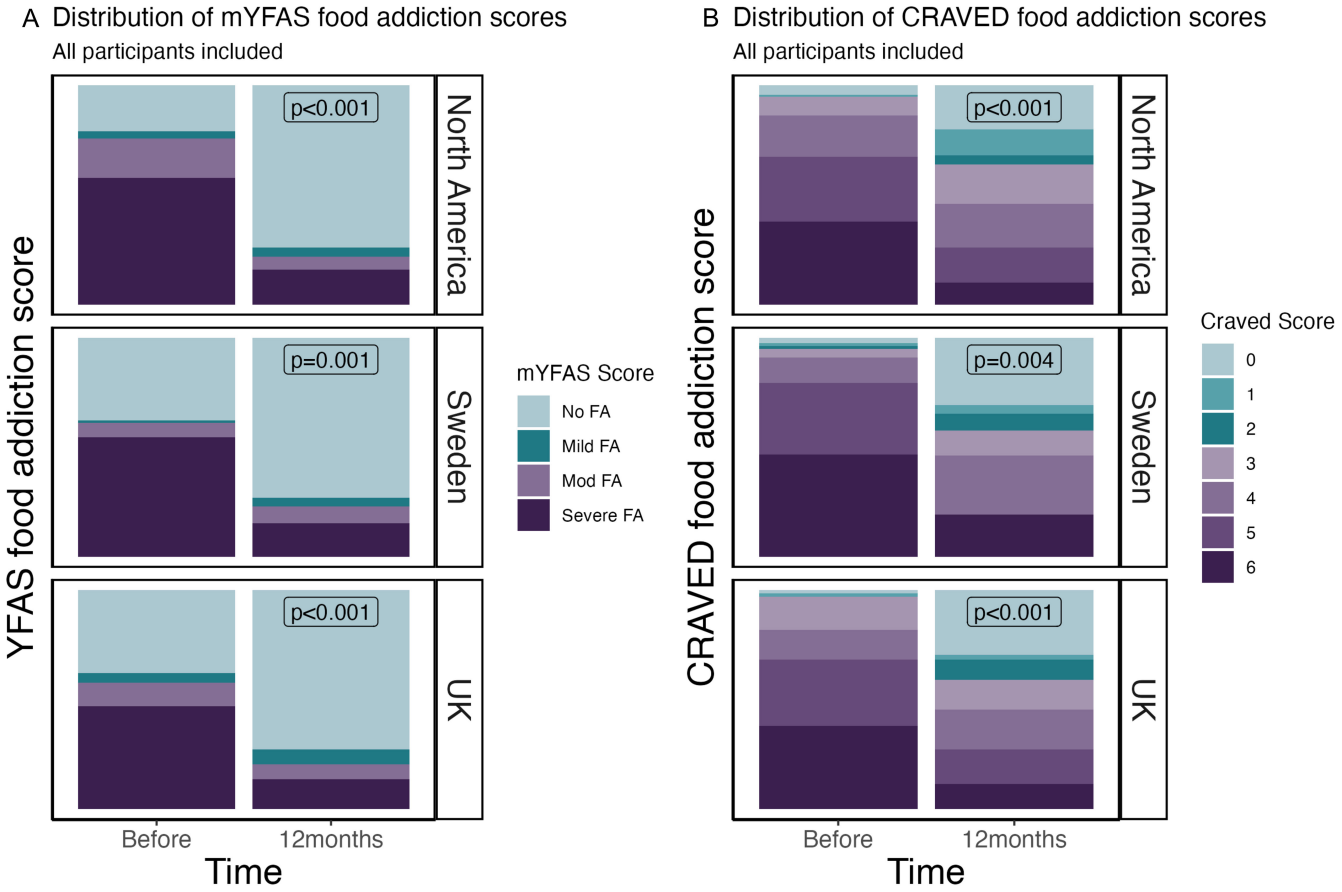
**(A)** mYFAS 2.0 severity indicators for participants in each location over time. **(B)** CRAVED severity indicators for participants in each location over time.

**Figure 2A** shows the change in mYFAS 2.0 severity indicators for participants in each location over time. There was a significant change from predominantly the ‘severe’ category to the ‘no food addiction’ category at 12 months across all locations (p<0.001). **Figure 2B** shows the reduction in CRAVED food addiction score at 12 months.

**Figure 3:**
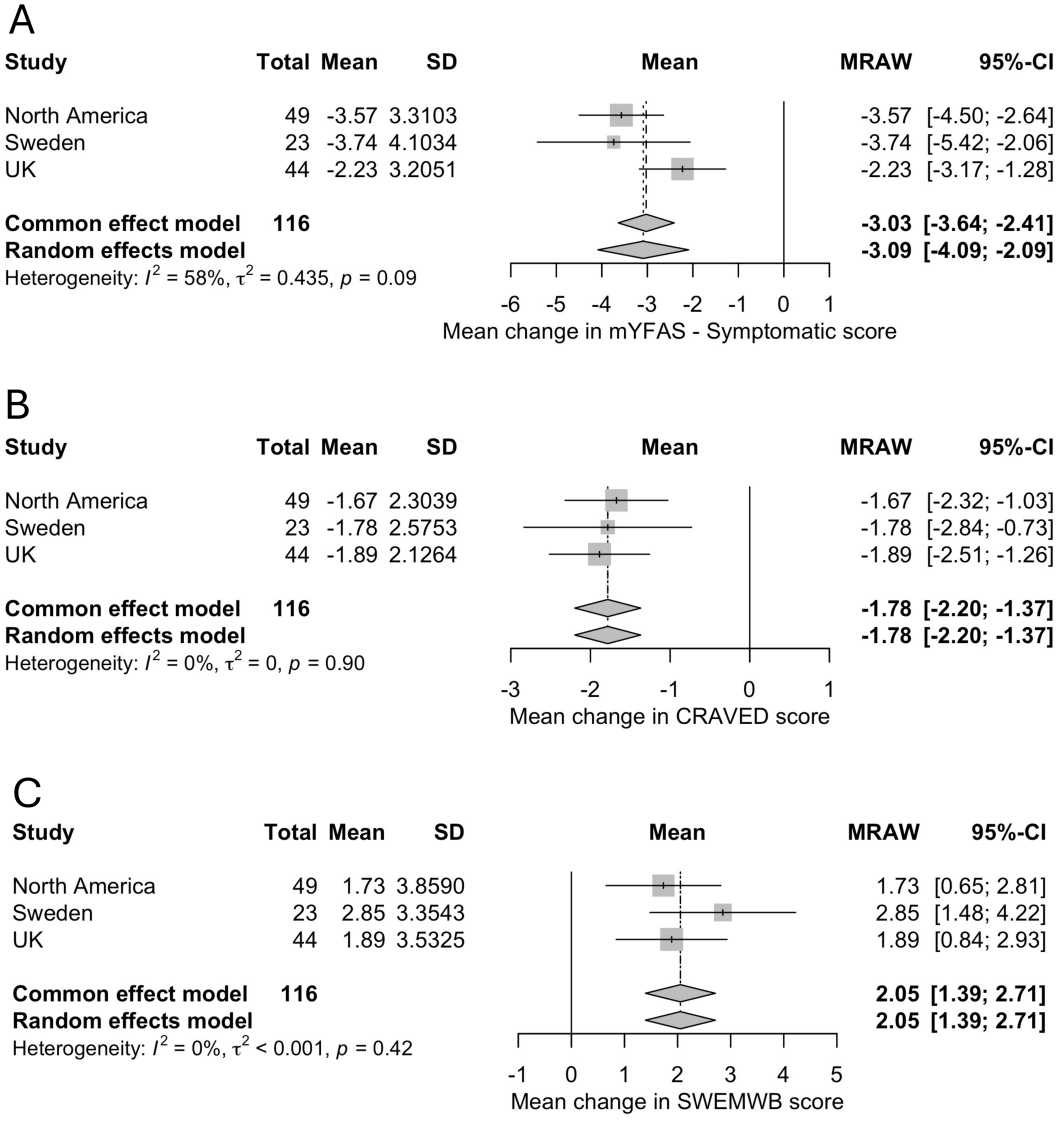
Summary statistics using forest plots. **(A)** mYFAS 2.0, **(B)** CRAVED, **(C)** SWEMWBS.

**Figure 3** shows summary statistics using forest plots. The data show that the aggregated results represent a significant reduction in food addiction symptoms (mYFAS 2.0 and CRAVED) and improvement in mental well-being (SWEMWBS) over the 12-month follow-up period.

## Discussion

There is a shortage of published data on intervention outcomes for individuals struggling with addictive behaviours relating to food. Even fewer studies report longer-term outcomes. Meanwhile, clinicians and coaches are actively providing services to clients seeking help. The data presented here represent the 12-month follow-up of an audit of similar online low-carbohydrate “real food” programs with psychoeducation and social support delivered across three locations in North America and Europe.

Across all three countries, participants independently set up support groups to share information, challenges, and successes between sessions. The interventions appeared accessible and acceptable to participants who provided data.

The significant long-term improvement in UPFA symptoms in both the mYFAS 2.0 and CRAVED is encouraging. The observation that individuals maintained improvements following the end of active treatment is noteworthy, given the known high relapse rates of other recognized addictive disorders (45).

High refined sugar and carbohydrate diets have been linked with poor mental health outcomes (25). Women with more refined carbohydrates in their diet were more likely to have depression 3 years later (46). Current participants’ mental well-being was lower than reported UK norms for the SWEMWBS pre-intervention (mean 23.5, SD 3.9) (38). However, post-intervention scores were similar to population norms. Improved well-being has measurable beneficial effects on health and quality of life (47).

Weight loss does not need to be a focus of UPFA treatment. Notably, 11.4% of people with UPFA are found normal- or underweight (48). Another study found that 5.5% of normal- and 15% of underweight people meet the criteria for UPFA (49). People of higher weights often seek UPFA treatment with the hope of weight reduction. This may explain why some ED professionals are skeptical of this field (8). The current data show that decreases in BMI were significant in two locations (UK and NA) despite not being a focus of the programs.

This audit has some limitations. Participants were predominantly female. Further studies are needed to establish suitable interventions for male individuals with UPFA. There is no control arm to compare participants who did not receive the intervention. Participants not completing the program and follow-up data likely had poorer outcomes than those completing the sessions (attrition bias). Furthermore, the intensive contact with clinicians and fellow participants can be therapeutic regardless of the nutrition intervention. The study did not include screening or measurement of EDs. It is known that UPFA and EDs often co-occur (16, 20, 22). Some of the outcome variability observed may be explained by considering this co-occurrence in future prospective studies.

The current data demonstrate the long-term clinical effectiveness of a low carbohydrate “real food” intervention delivered in an online group format with education and social support for individuals with UPFA symptoms. This intervention demonstrated clinically significant benefits for participants after 12 months. Larger, controlled and randomized intervention studies are needed to continue developing effective treatment protocols for this complex condition. It seems timely to compare an “abstinence-based” treatment approach described herein with the “all foods fit” (moderation) approaches among those with co-occurring UPFA and EDs, particularly binge-type EDs. Integration of UPFA-informed treatment models should be implemented with sensitivity to other models of ED recovery; clinicians and researchers from different schools of thought should join forces to improve patient care rather than oppose one another from deeply established intellectual allegiances.

## Data Availability

The raw data supporting the conclusions of this article will be made available by the authors, without undue reservation.
